# Supplementary material for: Content-rich biological network constructed by mining PubMed abstracts
Source: BMC Bioinformatics. 2004 Oct 8;5:147. doi: 10.1186/1471-2105-5-147 (PMC528731; doi:10.1186/1471-2105-5-147)
Supplement: Additional File 2 — The original results of the above study (non-essential files are deleted to keep the file size under the limit set by BMC bioinformatics). [file 1471-2105-5-147-S2.bz2 › chilibotAdditionalFile2/dip50/256ID8242751E1019/html/CDKN1A_CCNA2.html]

 


 **CDKN1A** and **CCNA2** 
  
Found 163 abstracts in PubMed, retrieved 50.  
 

 What does Google say? 
 PDF only 
| .edu only 

---

**Interactive relationship** (e.g. stimulation, inhibition, etc)

**Inhibitory relationship**- Furthermore, ethanol induced the expression of the cyclin dependent kinase Cdk inhibitor p21 waf1  [ **CDKN1A** ]  cip1  [ **CDKN1A** ] , and inhibited the induction of cyclin A  [ **CCNA2** ] .  Ref: 12079680 Eur J Pharmacol, 2002
- While the observed 10 fold inhibition of cyclin D1 cdk 4 associated kinase activity appeared independent of cdk inhibitors, the activity of cdk 2 decreased about 20 fold, reflecting joint effects of the lower abundance of its cyclin partners and a significant increase of the cdk inhibitor p21 CIP1  [ **CDKN1A** ]  WAF1  [ **CDKN1A** ] , which blocked the remaining cyclin A  [ **CCNA2** ]  E cdk 2 complexes.  Ref: 11463860 Mol Endocrinol, 2001
- We previously reported that overexpression of HSP25 delayed cell growth, increased the level of p21 waf  [ **CDKN1A** ] , reduced the levels of cyclin D1, cyclin A  [ **CCNA2** ]  and cdc2, and induced radioresistance in L929 cells.  Ref: 11965498 Cell Death Differ, 2002
- They also suggest that induction of p21 cip1  [ **CDKN1A** ]  by the viral protein NS1 arrests cells in G 2 through inhibition of cyclin A  [ **CCNA2** ]  dependent kinase activity.  Ref: 11602746 J Virol, 2001
- Also, CDK2L cyclin A  [ **CCNA2** ]  is inhibited to the same extent and by the same concentrations of p21 CIP1  [ **CDKN1A** ]  as CDK2 cyclin A  [ **CCNA2** ] .  Ref: 11506705 DNA Cell Biol, 2001
- p21 Waf1  [ **CDKN1A** ]  Cip1  [ **CDKN1A** ]  exerts a key role in driving this G 2 exit both by inhibiting cyclin B1 Cdk1 and cyclin A  [ **CCNA2** ]  Cdk1 2 complexes, which control G 2 M progression, and by blocking the phosphorylation of pRb family proteins.  Ref: 12881433 EMBO JEMBO J, 2001
- Since cyclin A  [ **CCNA2** ]  exists in the same quaternary complex in the S phase of the cell cycle as the cdk inhibitor p21WAF1 CIP1  [ **CDKN1A** ] , the levels of the two proteins were compared.  Ref: 11745687 J Pathol, 2001
**Stimulatory relationship**- Western blot analysis also shows that DHA greatly reduces the level of cyclin A  [ **CCNA2** ] , while increasing the level of p21 WAF1  [ **CDKN1A** ] , a cellular inhibitor of cyclin A  [ **CCNA2** ]  cyclin dependent kinase 2 activity.  Ref: 12492401 Biochem J< MedlineTA>Biochem J, 2001
- In cotransfection studies, cyclin A  [ **CCNA2** ]  Cdk1 inhibited CDP Cux stable DNA binding and prevented repression of the p21 WAF1  [ **CDKN1A** ]  reporter.  Ref: 11584018 J Biol Chem, 2001
- Thus, PD selectively elevates p21 WAF1  [ **CDKN1A** ]  CIP1  [ **CDKN1A** ]  levels and thereby arrests the cell cycle at G1 S phase by down regulating Cyclin A  [ **CCNA2** ]  Cdk2 activity.  Ref: 12819186 Carcinogenesis< MedlineTA>Carcinogenesis, 2001
**Neutral relationship**- Highly potent p21 WAF1  [ **CDKN1A** ]  derived peptide inhibitors of CDK mediated pRb phosphorylation delineation and structural insight into their interactions with cyclin A  [ **CCNA2** ] .  Ref: 12383116 J Pept Res, 2002
- In superficial spreading melanomas, the cyclin A  [ **CCNA2** ]  level was related to tumour thickness, with less expression in thinner lesions p< ; 0.00001, and to Ki 67 p< ; 0.00001 and p21WAF1 CIP1  [ **CDKN1A** ]  p=0.01 scores.  Ref: 11745687 J Pathol, 2001

**Non-interactive relationship** (e.g. studied together, co-existance, homology, etc.)

- METHODS Using cell culture and immunocytochemical techniques, we examined the cell growth, DNA synthesis, expression of PCNA, cyclin A  [ **CCNA2** ] , B 1, D 1, p16 ink4a and p21 cip  [ **CDKN1A** ]  waf1  [ **CDKN1A** ]  of SGC 7901 cells which were treated with various c9, t11 CLA concentrations 2 00 and 200 micromol.L 1 of c 9, t 11 CLA for 24 and 48h, with a negative control 0.1% ethane.  Ref: 11925596 World J Gastroenterol, 2002
- These results suggest that baicalein inhibits the proliferation of rat heart endothelial cells via G1 and G2 arrest in association with the down regulation of the expression and function of Cdk1, Cdk2, cyclin D2 and cyclin A  [ **CCNA2** ]  proteins, and up regulation of cyclin E, p15 Ink4B, p53 and p21 CIP1  [ **CDKN1A** ]  Waf1  [ **CDKN1A** ] .  Ref: 11513834 Eur J Pharmacol, 2001
- Baicalein mediated G1 and G2 growth arrest accompanied by the down regulation of cyclin D2, cyclin A  [ **CCNA2** ] , cyclin dependent kinase 1 Cdk1 and cyclin dependent kinase 2 Cdk2, and up regulation of p15 Ink4B, p21 CIP1  [ **CDKN1A** ]  Waf1  [ **CDKN1A** ] , p53 and cyclin E.  Ref: 11513834 Eur J Pharmacol, 2001
- In correlation with its antitumor activity both in vitro and in vivo, Compound 2 induces expression of p21 WAF1  [ **CDKN1A** ]  Cip1  [ **CDKN1A** ] , gelsolin, and keratin 19, while down regulating expression of cyclin A  [ **CCNA2** ]  and cyclin B1 in human cancer cells in a dose dependent manner.  Ref: 12154036 Cancer Res, 2002
- METHODS For each patient, flow cytometry DNA analyses on frozen samples and on immunohistochemical staining were performed, including Ki 67, cyclin A  [ **CCNA2** ] , p53, and p21 waf1  [ **CDKN1A** ]  p21, with assessment of the percentages of positive nuclei were assessed.  Ref: 12627500 Cancer, 2003
- Raf activation increased the expression of cyclin A  [ **CCNA2** ] , cyclin D, cyclin E, and p21 Cip1  [ **CDKN1A** ] , which are associated with G 1 progression.  Ref: 12429936 Cell Cycle< MedlineTA>Cell Cycle, 2003
- The array data enabled us to focus on the role of potential key genes e.g., GADD153, CHK2, p21 WAF1  [ **CDKN1A** ] , cyclin A  [ **CCNA2** ] , CDK1, and DHFR that might be targets of MSA in impeding cell cycle progression.  Ref: 12517777 Cancer Res, 2003
- Expression patterns of cell cycle and or apoptosis associated p27, p21 cip  [ **CDKN1A** ]  waf  [ **CDKN1A** ] , bax, bcl 2, cyclin A  [ **CCNA2** ]  and pro caspase 3 were studied using quantitative Western blotting.  Ref: 11830335 J Hepatol, 2002
- We show that NS1 mediated cell cycle arrest correlates with the accumulation of the cyclin dependent kinase Cdk inhibitor p21 cip1  [ **CDKN1A** ]  associated with both the cyclin A  [ **CCNA2** ]  Cdk and cyclin E Cdk2 complexes but in the absence of accumulation of p53, a potent transcriptional activator of p21 cip1  [ **CDKN1A** ] .  Ref: 11602746 J Virol, 2001
- During G1 phase arrest, the expression level of cyclin D1, cyclin A  [ **CCNA2** ] , and cyclin E was decreased, and the expression of CDK cyclin dependent kinase inhibitor, protein p21 Waf1  [ **CDKN1A** ]  Cip1  [ **CDKN1A** ] , was increased in a time dependent manner in a breast cancer cell MCF 7.  Ref: 12150857 Bioorg Med Chem, 2002
- Raf activation increased the expression of Cdk2, Cdk4, cyclin A  [ **CCNA2** ] , cyclin D, cyclin E, p21 Cip1  [ **CDKN1A** ]  and c Myc and decreased the expression of p27 Kip1 which are associated with G 1 progression.  Ref: 11466616 Oncogene, 2001
- When cells were synchronized in S phase with aphidicolin and then released, activation of Delta MEKK3 ER\* resulted in the up regulation of p21 CIP1  [ **CDKN1A** ]  and a pronounced inhibition of cyclin A  [ **CCNA2** ]  CDK2 and cyclin B1 CDK1 kinase activity.  Ref: 12444545 Oncogene, 2002
- In mammalian cells, CDK2 is part of a multiprotein complex that includes Cyclin A  [ **CCNA2** ]  or E and cell cycle regulatory proteins such as p21 Cip1  [ **CDKN1A** ] , PCNA, p27 Kip1, p45 SKP2, p19 SKP1, and CksHs1 CksHs2.  Ref: 11349131 J Biol Chem, 2001
- Panaxadiol selectively inhibits Cyclin A  [ **CCNA2** ]  associated Cdk2 activity by elevating p21WAF1 CIP1  [ **CDKN1A** ]  protein levels in mammalian cells.  Ref: 12819186 Carcinogenesis< MedlineTA>Carcinogenesis, 2001
- CONCLUSION The cell growth and proliferation of SGC 7901 cell is inhibited by c9, t11 CLA via blocking the cell cycle, with reduced expressions of cyclin A  [ **CCNA2** ] , B 1 and D 1 and enhanced expressions of CDKI P16 ink4a and p21 cip  [ **CDKN1A** ]  waf1  [ **CDKN1A** ] .  Ref: 11925596 World J Gastroenterol, 2002
- PS 341 exposure in H460 cells led to stabilization of p53, induction of p21 cip  [ **CDKN1A** ]  waf 1  [ **CDKN1A** ]  and MDM2 expression, an increase in cyclin B and cyclin A  [ **CCNA2** ] , and the activation of cyclin B and cyclin A  [ **CCNA2** ]  kinases.  Ref: 12631620 Clin Cancer Res, 2003
- TSA increased activation of caspase 3 and expression of cyclin A  [ **CCNA2** ] , p2l cip  [ **CDKN1A** ]  waf  [ **CDKN1A** ] , bax and pro caspase 3, while bcl 2 was downregulated.  Ref: 11830335 J Hepatol, 2002
- Similarly, levels of the p21 WAF1  [ **CDKN1A** ]  CIP1  [ **CDKN1A** ]  protein that is associated with the Cyclin A  [ **CCNA2** ]  Cdk2 complex increased, and these increases correlated well with the down regulation of Cyclin A  [ **CCNA2** ]  Cdk2 activity.  Ref: 12819186 Carcinogenesis< MedlineTA>Carcinogenesis, 2002
- However, activation of p42 44 MAPK in the above cells led to the inhibition of DNA synthesis, caused growth arrest, decrease in cyclin A  [ **CCNA2** ]  and upregulation of p21 Cip  [ **CDKN1A** ]  expression in a time dependent manner.  Ref: 12190111 Mol Cell Biochem, 2002
- Immunohistochemical expression of p53, p21 waf1  [ **CDKN1A** ] , rb, p16, cyclin D1, p27, Ki67, cyclin A  [ **CCNA2** ] , cyclin B1, bcl2, bax and bak proteins and apoptotic index in normal thymus.  Ref: 11642719 Histol Histopathol, 2001
